# Effectiveness of Ultrasound-Guided Canal Adductor Blockade for Chronic Pain and Functioning in Knee Osteoarthritis: A Prospective Longitudinal Observational Study

**DOI:** 10.1155/2022/5270662

**Published:** 2022-01-22

**Authors:** Mensur Salihovic, Boris Rijavec, Anida Muratagic, Rok Blagus, Urska Puh

**Affiliations:** ^1^Department of Anesthesiology and Surgical Intensive Care, University Medical Centre Ljubljana, Zaloška 2, 1000 Ljubljana, Slovenia; ^2^Emergency Medical Department, Novo Mesto, Kandijska cesta 4, Novo Mesto, Slovenia; ^3^Närhälsan Partille Health Centre, Kyrktorget 13, 433 33 Partille, Region Västra Götaland, Sweden; ^4^Institute for Biostatistics and Medical Informatics University of Ljubljana, Faculty of Medicine, Vrazov trg 2, 1000 Ljubljana, Slovenia; ^5^University of Ljubljana Faculty of Health Sciences, Zdravstvena pot, 1000 Ljubljana, Slovenia

## Abstract

**Methods:**

Seventy-seven patients with chronic knee osteoarthritis pain received ultrasound-guided ACB with 14 ml 0.25% levobupivacaine and 100 mcg clonidine. At baseline and 1 month after the blockade, we assessed maximal and minimal pain intensity in the knee using a numeric rating scale (NRS) and the Knee Injury and Osteoarthritis Outcome Score (KOOS). The range of motion in extension and flexion (ROMext and ROMflex) and quadriceps muscle strength of both knees (QS), Timed Up and Go Test (TUG), and 30-Second Chair Stand Test (30CST) results were determined at baseline, 1 hour, 1 week, and 1 month after the blockade.

**Results:**

ACB with levobupivacaine and clonidine appeared to decrease pain severity (NRS_max_ 8.13 to 4.2, *p* < 0.001 and NRS_min_ 3.32 to 1.40, *p* < 0.001). Similarly, knee ROMext decreased from 3.90 preintervention to 2.89 postintervention at 1 month, *p* < 0.001; ROMflex decreased from 5.70 to 3.29, *p* < 0.001; TUG time decreased from 3.22 to 2.93, <0.001; QS increased from 18.43 to 22.77, *p* < 0.001; CST increased from 8.23 to 10.74, *p* < 0.001. The KOOS for pain (36.40 to 58.34), symptoms (52.55 to 64.32), activities of daily living functions (ADLs, 36.36 to 60.77), and quality of life (QoL, 17.87 to 30.97) also increased, all *p* < 0.001.

**Conclusion:**

ACB appeared to decrease pain and increase ambulation. If our preliminary results are reproducible in a planned randomized controlled trial, ACB could be a useful adjunctive pain therapy in patients with disabling pain due to knee OA.

## 1. Introduction

The effects of adductor canal blockade on the control of acute postoperative pain in patients after total knee arthroplasty have been investigated in several studies [1, 2]. However, we could not find a reference regarding the effects of adductor canal blockade in patients with chronic knee OA pain or its influence on specific or overall body functioning. In some studies, nervous saphenous entrapment syndrome [3] and the composite effects of different painful knee states [4] were investigated.

Knee osteoarthritis (OA) is a progressive joint disease that is widely prevalent throughout the world [5]. An annual incidence of up to 6% makes knee OA one of the greatest current health and financial burdens on patients globally [5]. Women are more affected than men; they report greater pain and have more radiographic changes, impairments of body functions, and limitations of activities [5]. Knee OA is characterized by chronic pain and significantly reduces patient functioning and quality of life. Up to 10% of knee OA patients are unable to perform daily activities [5]. The etiology of OA is not well understood. Repetitive injuries, especially during extreme physical activities, e.g., specific sports or occupations that require frequent squatting and kneeling, often lead to knee OA [6]. Similar to rheumatoid arthritis and posttraumatic arthritis disease, knee OA is initially more prominent on one side of the body and later causes changes on both sides, presenting as a chronic bilateral inflammatory disease [7, 8]. Knee OA pain correlates with changes in neural activity and with histologic changes, indicating that the sprouting of sensory and sympathetic nerves in damaged structures of the knee joint could be a dominant factor in these processes [7, 8].

In the early stages of OA, various approaches offer satisfactory effectiveness for pain reduction and functional improvement, e.g., nonsteroidal anti-inflammatory drugs, paracetamol (acetaminophen), metamizole, and physical therapy [9]. However, many elderly patients have relative or absolute contraindications for nonsteroidal anti-inflammatory drugs [10]. In advanced OA, the effectiveness of nonopioid analgesics and physical therapy for pain and functioning decreases, and eventually, only total knee arthroplasty can alleviate unbearable chronic knee pain and improve functioning and quality of life [11]. Recently published studies show promising results with the use of radiofrequency therapy [12]. However, many patients refuse surgery because of their beliefs, fears of anesthesia or surgery outcomes, or severe concomitant diseases that make them *high-risk patients* for such treatment. Furthermore, a remarkable number of patients continue to have postoperative chronic knee pain [13].

The objective of this study was to investigate the effects of adductor canal blockade with local anesthetic levobupivacaine and central *α*₂-adrenergic agonist clonidine on knee pain and improvement of overall functioning in patients with chronic OA knee pain. Pain intensity, assessed by a numerical rating scale (NRS), was used to measure the primary outcome. We hypothesized that pain would decrease after the blockade, especially in the blocked knee. Furthermore, we hypothesized that passive knee range of motion (ROM) and quadriceps muscle strength (QS) would increase at the blocked knee, and that functional performance tests and the patients' perception of the knee and associated problems would improve.

## 2. Methods

### 2.1. Participants

A prospective, observational, longitudinal, noncontrolled study was performed. A convenience sample of patients who had been referred by their family physician to the outpatient pain clinic was recruited consecutively. Using a two-sided alpha equal to 0.05 and assuming that the standard deviation at each time point is equal to 4 and that the correlation between the two pairs of measurements is equal to 0.1, it was estimated that 77 patients would be needed to obtain 80% power to detect a mean difference of 1.8 between the two pairs of measurements. The study was approved by the Ethics Committee of Republic Slovenia (KME 100/02/15) and retrospectively registered at ClinicalTrials.gov (NCT02695654). After receiving information about the study and potential risks, all patients provided written informed consent. The inclusion criteria were a minimum age of 50 years, chronic pain in the knee that started at least 6 months before the study (NRS > 5), confirmed diagnosis of knee OA, sufficient cognitive function to understand study procedures, and the ability to communicate with site personnel. None of the subjects previously experienced sufficient therapeutic effects from other treatments, such as intra-articular injection of hyaluronidase or intraarticular blockade, physical therapy, acupuncture, and nonopioid or opioid drugs. Most of the patients had already been diagnosed with knee OA, but in each case, the diagnosis was independently confirmed by the orthopedic surgeon on our research team. The American College of Rheumatology clinical classification criteria for OA of the knee (history, physical examination, and radiographic findings, plus their diagnostic criteria for knee OA: pain in the knee and one of the following: age over 50 years, less than 30 minutes of morning stiffness, crepitus on active motion, and osteophytes) were used [14]. All patients included in our research fulfilled both criteria for idiopathic OA: clinical ACR and grade 2 or higher of Kellgren and Lawrence classification [15].

The exclusion criteria were any cardiovascular, hepatic, or renal condition that would compromise participation; severe neurologic conditions; opioid dependency (opioid intake for more than 3 months and more than 30 mg daily oral morphine equivalent); coexisting severe hematological disorder or derangement of coagulation parameters; psychiatric illnesses; allergy to any of the drugs used in the study; infection or malignancy; and active systemic infection. Decisions regarding whether a patient fulfilled the inclusion and exclusion criteria for the study were made by the anesthesiologist.

### 2.2. Outcome Measures

Maximal and minimal pain intensity in the blocked knee in the previous week was assessed by the anesthesiologist using an 11-point verbal NRS scale (NRS_max_ and NRS_min_) prior to baseline and 1 month after the intervention. The subjects were asked to indicate the single number on a scale from 0 to 10 that best rated the intensity of their maximal and minimal pain in the knee. The two extreme categories of the scale were labeled “no pain” and “the worst imaginable pain” [16]. Some assessments (ROM, QS, functional performance tests, and related NRSs) were performed four times: at baseline, 1 hour, 1 week, and 1 month after the intervention. To reduce fatigue and the duration of the assessment, other assessments (NRS for the previous week and Knee Injury and Osteoarthritis Outcome Score—KOOS) were performed only twice: at baseline and 1 month.

Additionally, immediately after the knee joint flexion range of motion (ROM) and quadriceps muscle strength (QS) measurements and performance-based tests were conducted by one of the three physical therapists, the participants were asked to rate their pain during the preceding test using the verbal NRS. The verbal NRS-11 for the assessment of osteoarthritic knee pain has moderate [17] to excellent test-retest reliability [16].

Passive knee joint ROM of extension and flexion was measured in the control and blocked leg in the supine position using a goniometer and considering the bony landmarks of the greater trochanter, lateral femoral condyle, and lateral malleolus. Maximum voluntary isometric contraction of the quadriceps muscle of each leg was measured with a dynamometer (Model-01163, Lafayette Instrument Company, USA). The participants were seated on a treatment table with the hips and knees in 90° flexion. The dynamometer was stabilized with a belt attached to the table and around the subject's leg. Its pad was placed perpendicular to the tibial crest, three finger widths (5 cm) proximal to the ankle joint line. The participants were instructed to press as forcefully as possible against the dynamometer by gradually increasing the force over a period of two seconds until the maximum was reached and to continue their maximal effort for another three seconds, i.e., until the device beeped a second time. After one trial for familiarization (more were provided if needed) and warm-up, three measurement trials separated by 30 seconds of rest were performed. A rater gave standardized verbal encouragement during each trial. Peak forces were recorded. If the difference between the three trials exceeded 10%, an additional trial was performed [18]. The mean of the three best trials was used for further analyses. ROM and QS measurements were performed at all four assessment sessions. Excellent interrater reliabilities of knee ROM measurements in flexion and extension have been reported for patients with knee OA [19]. QS assessment using beltstabilized handheld dynamometry has shown excellent intrarater reliability in patients with knee OA [20].

Two performance-based tests, the Timed Up and Go Test (TUG) and the 30-Second Chair Stand Test (30CST), were performed at all four assessment sessions. Both tests are recommended standardized tests for assessing physical function in people with knee OA [21]. In the TUG, the time needed for the subject to stand, walk 3 m, turn, return, and sit on a chair is measured. Participants are instructed to walk as quickly as they safely can. Following a practice trial, two measurement trials were performed, and the mean of these trials was used for further analyses. In the 30CST, number of times the participant rises from a chair in 30 s is counted. The TUG has shown good reliability in patients with hip and knee OA [22] and excellent intra- and interrater reliability in patients with mild to moderate knee OA [23]. For the 30CST in patients with knee OA, excellent intrarater reliability [20, 24] has recently been reported. The TUG and sit-to-stand tests are selective and functionally valid for patients with knee OA [25].

The Slovenian translation of the KOOS (http://www.koos.nu/) was administered at baseline and 1 month following the intervention. The KOOS is a self-reported 5-subscale questionnaire that collects patients' opinions about their knee and associated problems. In this study, 4 subscales (pain, other symptoms, activities of daily living (ADLs), and knee-related quality of life (QoL)) were assessed. The sports and recreation subscale was not used since multiple studies have reported its floor effect [26] and because we assumed that it was not relevant to our sample because of the participants' age. Using a 5-point Likert scale, each subscale is scored separately from 0 (extreme knee problems) to 100 (no knee problems). The KOOS has shown adequate internal consistency, good test-retest reliability, and construct validity and responsiveness in young and older adults with knee injuries and/or OA [26].

In addition, at baseline, the participants were asked the following question: “What is the least amount of any improvement that the treatment would have to achieve in one month for you to conclude that it was worthwhile and that you are satisfied?” One month after the intervention, the participants indicated whether that goal had been achieved by answering “yes” or “no.”

### 2.3. Intervention

All patients were required to fast before the procedure. We also request that our patients do not take their usual analgesic medication on the day of the blockade. We also request that they continue with their daily routine also during a study and help themselves as usually, including drugs or exercises. We requested from them that they don't use new analgesics or physiotherapy, or any new kind of therapy that could potentially impact their pain.

The block was performed in the operating room with the patient lying in the supine position on the table, and all patients were monitored for blood pressure, pulse oximetry (SpO2%), and electrocardiography during the procedure. After sterile surgical preparation of the block site, blockade of the adductor canal was performed under ultrasound control by the same trained anesthesiologist. To perform the blockade, we used an 8- or 10 cm 20G echogenic needle (Stimuplex Ultra 360 Braun), and a 12- to 18 MHz linear probe with a Flex focus 800 Analogic BK Medical ultrasound device was used as background to perform the block. After the pilot phase, we decided to use an approach similar to one recommended in a recently published anatomic study of knee innervation, specifically that of the anterior capsule of the knee and the adductor canal [27]. We targeted the anterolateral area of the adductor canal and femoral vessels [27] using 14 ml of 0.25% levobupivacaine and 100 mcg clonidine mixed in the same syringe. The puncture site was the middle of the adductor canal, which was estimated by scanning the medial thigh.

According to the mentioned study [27], this provides the best probability of encompassing both the main nerves of the adductor canal, the saphenous nerve, and the nerve to the vastus medialis. We regularly checked the block using alcohol/dry swabs and followed up on any sign of side effects.

After confirming the effect of the blockade, we continued with the study process as previously described, and the patient was discharged to home after one hour of monitoring of SpO2% and other vitals as necessary in the recovery room.

### 2.4. Statistical Analysis

Data are presented as frequencies (%) or means and standard deviations (SDs) as appropriate. To test for differences between male and female patients in the demographic characteristics at baseline, we used Fisher's exact test or the Welch two-sample *t*-test, as appropriate. The other data were analyzed with linear or Poisson mixed effects models for continuous and count data, respectively. To account for multiple measurements in each patient, a random intercept by patient ID was included in the model. A square root transformation was applied when necessary to meet the assumption of normality of the model residuals. A *p* value < 0.05 was considered statistically significant; all 95% confidence intervals (CIs) were two-sided. R statistical language (R version 3.6.1.) was used for the analyses [28]; the mixed effects models were estimated using the R package lme4 [29].

## 3. Results

### 3.1. Participants

The study included 77 participants (female 83%). Their ages ranged from 47 to 92 (mean 66.7 and SD 12.8), and they were slightly obese on average (mean BMI 30.8 and SD 5.4). Seventy-five participants finished the study. One participant dropped out and did not complete the 1-month assessment. Another participant dropped out and did not complete the 1-week and 1-month assessments. All blockades were successfully performed, and we did not observe any notable side effects.

### 3.2. Body Function and Structure Assessments

Maximal and minimal pain intensity in the previous week decreased significantly after the intervention (Table 1). The estimated adjusted effect sizes (ES) were large.

The extension and flexion ROM values at the control knee were similar in all the measurements. In contrast, the ROMs at the blocked knee increased significantly in both directions in the later measurements when compared with the baseline, thus decreasing the difference between the control and blocked limbs (Table 2).

Pain intensity during flexion ROM measurement was significantly lower in the later measurements than at baseline in both knee joints. However, the difference between the two knee joints was the lowest 1 hour after the intervention and remained similar at the later measurements (Table 2).

QS increased significantly 1 hour after the intervention for the blocked knee but not for the control knee, thus reducing the difference between the two limbs 1 hour after baseline compared with the difference at baseline. However, QS increased similarly in both limbs at the 1-week and 1-month measurements compared with baseline and thus did not affect the difference between the blocked and the control knee at those time points versus at baseline (Table 3).

Pain during QS measurement decreased significantly in both knee joints at the later measurements compared with baseline. The difference between the two limbs decreased 1 hour after the intervention compared with the difference at baseline. The difference then increased at the later measurements, reaching a slightly higher level than at baseline (Table 3).

### 3.3. Activity and Participation Assessments

The time needed to perform the TUG decreased 1 hour after the intervention, achieved its minimum at the 1-week measurement, and remained at a similar point at the 1-month measurement (Table 4). Similarly, the number of repetitions on the 30CST increased over time, achieving the largest value at the 1-month measurement. All the estimated adjusted effect sizes were large.

Pain intensity during the TUG and the 30CST decreased substantially 1 hour after the intervention and then remained at similar (although slightly higher) levels in the later measurements.

KOOS questionnaire scores for pain, symptoms, ADLs, and QoL increased significantly 1 month after the intervention compared with the baseline measurement (Table 5). The estimated adjusted effect sizes were large.

Regarding satisfaction with the outcome of the intervention/achievement of the goal, 58 (77%, 95% CI: 66-86%) participants answered “yes.”

## 4. Discussion

In patients with knee OA, pain is a dominant symptom and the cause of significant functional limitation; therefore, it must be properly controlled [5, 11]. The results of our study showed that pain control was possible to some degree. After the intervention, all pain assessments showed a significant reduction in pain intensity in the blocked knee compared to the baseline. Pain reductions exceeded the minimum detectable change (MDC) of 1.33 [16] in NRS_max_ and NRS_min_ in the previous week (1-month assessment) and during passive knee ROM, QS measurements, and both functional performance tests at all three postintervention assessments. In contrast, the changes in pain intensity during ROM and QS measurements for the control knee were lower than the MDC [16] and lower than the standardized response mean (SRM) of 1.15 reported after total knee replacement [17]. As expected, the differences in pain intensity between the two knee joints during these measurements were the lowest 1 hour after the intervention. However, for both knee joints, the relative ESs of pain reduction during ROM and QS measurements were moderate and low at the 1-month QS measurements. Bilateral changes after blockade are consistent with the mostly bilateral nature of the illness [30] and with the established pathologic reflexes or quadriceps arthrogenic muscle inhibition (AMI), which are presumed to typically be bilateral [30–32].

We assume that the adductor canal blockade decreased nociceptive signaling from the knee and pain signal input at the spinal cord level. Due to prolonged effect of LA, we added clonidine, counting on extended duration of peripheral desensitization. [33]. This decrease in pain signal input led to the disinhibition of the motor portion of the sensory-motor reflex arch at the spinal cord, resulting in easier muscle contraction and an increase in QS [34, 35], which we also confirmed in this study. In fact, most of the effects of the blockade could be attributed to the change in AMI [32]. Spinal reflex pathways that could contribute to AMI include the gamma-loop, flexion reflex, and group I nonreciprocal (Ib) inhibitory pathways [35, 36]. The further influence of changes in the ascendant and descendant pain control pathways may lead to the resolution of neuroplastic changes in the central nervous system, which are often present in patients with chronic pain [35, 37]. Finally, in some cases, this may result in the resolution of central sensitization, which is also often present in many patients with chronic pain, regardless of its source [37, 38]. We assume that the significant reduction in pain due to the blockade not only influenced the patients' ability to perform the functional tests 1 hour after the blockade but also led to increased mobility during daily activities, which may have had a further positive influence on decreasing pain and improving the functional test outcomes (at 1 week and 1 month) and KOOS. It is possible that the patients' ability to exercise was increased, and they were able to exercise more easily and perhaps more often than before the blockade. All this could further improve the test results, given a previously reported strong negative correlation between pain and QS [39]. The results of the performance-based tests, the TUG and 30CST, improved significantly at the 1-week and 1-month measurements, respectively.

The TUG changes exceeded the MDC of 1.10 seconds in patients with mild to moderate knee OA [23] but not the MDC of 2.49 seconds in patients with hip and knee OA [22]. The TUG is used to assess global mobility function, but if performed with a fast walking speed, it can also test dynamic balance control. It is important to note that dynamic balance did not decrease 1 hour after the blockade, which could reflect the negative consequence of a possible decrease in proprioception from the knee area. The largest ES (0.33) was calculated at 1 month, similar to a previous study [40] of knee OA patients who underwent exercise-based physical therapy. A moderate correlation of the TUG with pain intensity (*r* = 0.58) has been reported [25]. Sit-to-stand tests are used to identify how a patient's knee function is affected [25]. In our study, the MDC of 2.4 stands [24] reported for the 30CST in patients with knee OA was exceeded only at 1 month. As expected, this outcome was still lower than normal reference values [41]. Clinically important improvements in the pain, symptoms, and ADL subscales of the patient-reported outcome measure, the KOOS, were also clinically important; [40, 42] confirmed improvement in patients' overall ability 1 month after the intervention and significant changes in the other outcomes. Additionally, the change in the QoL subscale was statistically significant, but it did not exceed the MCID [40]. In our study, the effect sizes of all four KOOS subscales were large; however, as expected for such a chronic condition, the scores after the intervention were still much lower than the reference data for healthy subjects (Pain: 91.0-95.6; Symptoms: 87.8-93.3; ADL: 92.4-98.0; QoL: 80.9-89.5) [43]. The changes in the QoL subscale indicate the possible influence of pain reduction on improvement in this dimension. The minimal clinically important differences (MCIDs) for the KOOS subscales in patients who have undergone posttotal knee replacement rehabilitation are 16.7 for pain, 10.7 for symptoms, 18.4 for ADLs, and 15.6 for QoL [40]. Additionally, the NRS and KOOS results in our study were strongly positively correlated with responsiveness to blockade, a result that is aligned with the conclusions of a meta-analysis indicating that NRS and VAS, as patient reported outcomes, are positively correlated with responsiveness to treatment [42]. In patients with knee OA, moderate to good negative correlations (*r* = −0.52 to -0.66) of the KOOS subscales with the TUG were reported [42]. Additionally, the high rate of patient satisfaction with the achievement of the treatment goal (77%) suggests the clinical acceptability of adductor canal blockade.

There are some limitations of this study. The main one is that there was no placebo control. We based our decision on the known limitations of placebo control in pain studies. First, it is possible that even long-term pain reduction is due to the placebo effect, which suggests that the placebo effect cannot be completely eliminated [44]. Second, the choice of a methodology to account for the placebo effect is further complicated by the fact that generally, contextual placebo cannot be eliminated in a study such as this one [44]. Third, we would have need to include a sham treatment in our study, which is somewhat challenging to perform and interpret. Indeed, needle penetration produces an important body response that is not easy to interpret, e.g., sham can impact the diffuse noxious inhibitory control system (DNIC) [44]. The DNIC or conditioning maintained pain system (CMP) has been proven to be significantly changed in people with chronic knee OA pain in some studies [44]. A further limitation is that there was no comparison group. Any study design using a comparison group (e.g., one treated with physiotherapy and/or pharmaceutical intervention) would require a significantly longer study term because we wanted to recruit patients with an advanced stage of knee OA. We would assume that most patients with advanced knee OA have tried recommended pharmaceutical/physiotherapy interventions and have already found that such therapy as not able to improve either pain or ability. It is very unlikely that these patients would choose to participate in a study in which they might receive a treatment that they have previously had no success with. Furthermore, it would take a very long time to recruit a significant number of such patients, which would also increase the chances of technical problems such as patient dropout [44]. Next limitation of our study is short lasting. The decision was based on conceivable results in pilot phase of study, and our aim to prove that the results are good enough to proceed with the controlled study. However, despite their disadvantages, placebo-controlled studies are the best way to determine the importance of a phenomenon. It is accepted that prospective noncontrolled studies are useful for showing that a phenomenon warrants further examination in placebo-controlled studies [44]. In our opinion, most of our results had large effect sizes, which justifies future studies with a placebo-controlled design.

## 5. Conclusion

In our study, we found that ultrasound-guided adductor canal blockade was associated with significantly improved functionality and pain measurement results in patients with knee OA. This predominantly sensory block could provide patients with improved mobility, thereby further facilitating muscle strengthening, diminishing pain, improving mobility, and perhaps improving quality of life. This study showed that adductor canal blockade is a harmless approach that is well tolerated by patients exposing this procedure as safe and with a great potential to improve symptoms in patients with knee OA. It may be especially useful for high-risk patients with contraindications for anesthesia and/or surgery or those who refuse surgery for various reasons. The results of this study highlight the possible influence of decreasing nociceptive input using blockades, which may diminish pathologic reflexes, as a way to address different chronic pain states. But to prove its real effectiveness, further controlled study is needed.

## Figures and Tables

**Table 1:** Maximal and minimal pain intensity in the blocked knee at baseline and 1 month after adductor canal blockade and their differences with estimated adjusted absolute effect size (*n* = 75).

	Assessment time	Mean (SD)	Effect size^+^	95% CI	*p* value
NRS_max_	B/L	8.13 (1.22)			
	1 M	4.2 (2.20)	-3.93	-4.46--3.41	<0.001

NRS_min_	B/L	3.32 (2.11)			
	1 M	1.40 (1.52)	-1.92	-2.38--1.46	<0.001

NRS: numeric rating scale; B/L: baseline; 1 M: 1 month after adductor canal blockade; ^+^age-, sex-, and BMI-adjusted effect size, as estimated by the linear mixed effects model.

**Table 2:** Changes in passive knee range of motion and related pain intensity over time, with estimated adjusted effect size.

Time, leg	Mean (SD), *n*	Effect size^+^	95% CI	*p* value	Effect^Ͱ^ size	95% CI	*p* value
ROM extension (°)							
B/L, control	0.84 (3.92), 77						
B/L, blockade	3.90 (5.99), 77	3.22	2.32–4.12	<0.001			
1 H, control	0.97 (3.89), 77				0.13	-0.76–1.03	0.773
1 H, blockade	3.05 (4.94), 77	2.24	1.34–3.14	<0.001	-0.86	-1.74–0.04	0.061
1 W, control	1.35 (4.08), 74				0.47	-0.43–1.38	0.311
1 W, blockade	2.97 (4.96), 74	1.78	0.86–2.70	<0.001	-0.98	-1.88--0.08	0.035
1 M, control	1.06 (3.73), 72				0.25	-0.65–1.17	0.587
1 M, blockade	2.89 (4.49), 72	1.83	0.91–2.76	<0.001	-1.14	-2.04--0.24	0.014
ROM flexion (°)							
B/L, control	127.17 (13.20), 77						
B/L, blockade	119.90 (14.67), 77	-7.43	-9.55--5.32	<0.001			
1 H, control	127.53 (13.22), 77				0.37	-1.74–2.49	0.733
1 H, blockade	125.61 (11.20), 77	-2.08	-4.19–0.04	0.054	5.72	3.60–7.84	<0.001
1 W, control	128.31 (12.56), 74				1.20	-0.94–3.34	0.272
1 W, blockade	125.58 (11.84), 72	-2.77	-4.93--0.61	0.012	5.87	3.72–8.01	<0.001
1 M, control	128.50(12.39), 72				1.69	-0.45–3.85	0.122
1 M, blockade	124.86 (12.67), 72	-3.64	-5.81--1.47	0.001	5.49	3.33–7.65	<0.001
Pain at ROM flexion measurement							
B/L, control	2.19 (2.62), 77						
B/L, blockade	5.70 (2.56), 77	2.63	2.20–3.14	<0.001			
1 H, control	1.62 (2.25), 77				0.74	0.58–0.93	<0.001
1 H, blockade	3.04 (2.48), 77	1.90	1.53–2.37	<0.001	0.53	0.45–0.63	<0.001
1 W, control	1.51 (2.03), 74				0.70	0.55–0.89	<0.001
1 W, blockade	3.20 (2.11), 74	2.12	1.69–2.65	<0.001	0.57	0.48–0.67	<0.001
1 M, control	1.56 (2.34), 72				0.69	0.54–0.88	<0.001
1 M, blockade	3.29 (2.51), 72	2.12	1.69–2.65	<0.001	0.56	0.47–0.66	<0.001

ROM: range of motion; B/L: baseline; 1 H: 1 hour; 1 W: 1 week; 1 M: 1 month after the adductor canal blockade; ^+^age-, gender-, and BMI-adjusted absolute (relative) effect size for the difference between the blockade and control limbs at different measurements, as estimated with the linear (or Poisson) mixed effects model including the interaction between measurement and limb. A Poisson mixed effects model was used for pain at ROM flexion measurement; age-, sex-, and BMI-adjusted absolute (relative) effect size for the difference between 1 hour, 1 week, and 1 month versus baseline for the blockade and control limbs, as estimated with the linear (or Poisson) mixed effects model including the interaction between measurement and limb. A Poisson mixed effects model was used for pain at ROM flexion measurement; age-, sex-, and BMI-adjusted absolute (relative) effect size for the difference between 1 hour, 1 week, and 1 month versus baseline for the blockade and control limbs, as estimated with the linear (or Poisson) mixed effects model including the interaction between measurement and group.

**Table 3:** Changes in quadriceps muscle strength and related pain intensity over time, with estimated adjusted effect size.

Time, leg	Mean (SD), *n*	Effect size^+^	95% CI	*p* value	Effect^Ͱ^ size	95% CI	*p* value
QS (kg)							
B/L, control	20.33 (8.64), 77						
B/L, blockade	18.43 (9.69), 77	-1.86	-3.07--0.64	0.003			
1 H, control	21.26 (8.14), 77				0.88	-0.33–2.11	0.155
1 H, blockade	20.38 (8.33), 77	-0.85	-2.07–0.37	0.170	1.89	0.67–3.11	0.002
1 W, control	24.09 (8.80), 73				3.83	2.59–5.08	<0.001
1 W, blockade	22.51 (9.71), 74	-1.71	-2.95--0.46	0.007	3.98	2.74–5.22	<0.001
1 M, control	24.12 (8.87), 71				4.08	2.83–5.33	<0.001
1 M, blockade	22.77 (9.39), 72	-1.52	-2.77--0.26	0.018	4.42	3.18–5.67	<0.001
Pain at QS measurement							
B/L, control	1.95 (2.37), 77						
B/L, blockade	4.47 (2.79), 77	2.29	1.89–2.77	<0.001			
1 H, control	1.55 (2.17), 77				0.79	0.61–0.89	<0.001
1 H, blockade	2.16 (2.24), 77	1.42	1.12–1.80	<0.001	0.79	0.40–0.59	<0.001
1 W, control	1.00 (1.67), 73				0.52	0.39–0.68	<0.001
1 W, blockade	2.58 (2.23), 74	2.18	1.63–2.91	<0.001	0.57	0.47–0.69	<0.001
1 M, control	0.93 (1.62), 71				0.47	0.35–0.63	<0.001
1 M, blockade	2.04 (2.30), 72	2.53	1.94–3.31	<0.001	0.45	0.36–0.55	<0.001

QS: quadriceps muscle strength; B/L: baseline; 1 H: 1 hour; 1 W: 1 week; 1 M: 1 month after the adductor canal blockade; ^+^age-, gender-, and BMI-adjusted absolute (relative) effect size for the difference between the blockade and control limbs at different measurements, as estimated with the linear (or Poisson) mixed effects model including the interaction between measurement and limb. A Poisson mixed effects model was used for pain at QS measurement; ^Ͱ^age-, gender-, and BMI-adjusted absolute (relative) effect size for the difference between 1 hour, 1 week, and 1 month versus baseline for the blockade and control limbs, as estimated with the linear (or Poisson) mixed effects model including the interaction between measurement and group.

**Table 4:** Changes in the functional performance tests and related pain intensity over time with estimated adjusted effect size.

	Assessment time	Mean (SD), *n*	Effect size^+^	95% CI	*p* value
TUG (s)^1/2^					<0.001
	B/L	3.22 (0.92), 77			
	1 H	3.10 (0.82), 76	-0.14	-0.19--0.04	0.004
	1 W	2.93 (0.68), 73	-0.30	-0.37--0.22	<0.001
	1 M	2.93 (0.68), 72	-0.33	-0.40--0.26	<0.001

Pain at TUG					<0.001
	B/L	4.53 (2.40), 77			
	1 H	1.83 (2.16), 76	0.41	0.33–0.50	<0.001
	1 W	2.40 (2.11), 73	0.53	0.44–0.63	<0.001
	1 M	1.94 (2.20), 72	0.42	0.34–0.52	<0.001

					<0.001

30CST (*n* repetitions)	B/L	8.23 (3.97), 77			
	1 H	9.36 (3.84), 76	1.11	0.68–1.55	<0.001
	1 W	10.33 (4.15), 73	2.12	1.68–2.57	<0.001
	1 M	10.74 (4.45), 72	2.64	2.20–3.08	<0.001

Pain at 30CST					<0.001
	B/L	5.68 (2.34), 75			
	1 H	2.82 (2.31), 73	0.50	0.42–0.59	<0.001
	1 W	3.34 (2.16), 70	0.59	0.50–0.70	<0.001
	1 M	3.32 (2.39), 69	0.58	0.49–0.69	<0.001

TUG: Timed Up and Go Test; 30CST: 30-Second Chair Stand Test; B/L: baseline; 1 H: 1 hour; 1 W: 1 week; 1 M: 1 month after the adductor canal blockade; ^+^age-, gender-, and BMI-adjusted effect size as estimated by the linear or Poisson mixed effects model as appropriate. For TUG, a square root transformation was applied. A Poisson mixed effects model was used for pain at TUG and 30CST.

**Table 5:** Knee Injury and Osteoarthritis Outcome Scores at baseline and 1 month after adductor canal blockade and their differences with estimated adjusted effect sizes.

KOOS	Assessment time	Mean (SD), *n*	Effect size^+^	95% CI	*p* value
Pain	B/L	36.40 (14.43), 75			
	1 M	58.34 (18.86), 73	21.90	17.96–25.84	<0.001

Symptoms	B/L	52.55 (18.14), 75			
	1 M	64.32 (18.28), 74	11.78	7.85–15.70	<0.001

ADLs	B/L	36.36 (15.96), 75			
	1 M	60.77 (19.47), 74	24.39	19.91–28.87	<0.001

QoL	B/L	17.87 (11.80), 75			
	1 M	30.97 (20.25), 73	13.13	8.64–17.62	<0.001

KOOS: Knee Injury and Osteoarthritis Outcome Score; ADLs: activities of daily living subscale; QoL: quality of life subscale. ^∗^The sports/recreation subscale was not administered; B/L: baseline; 1 M: 1 month after adductor canal blockade; ^+^age-, gender-, and BMI-adjusted effect size, as estimated by the linear mixed effects model.

## Data Availability

The minimal dataset required to replicate the reported study findings are within the paper. Additional data cannot be shared publicly because of potentially sensitive patient information. A nonauthor contact that interested researchers can get in touch with in terms of accessing the data is Dr. Peter Poredos, Head of the Department of Anesthesiology and Surgical Intensive Care, University Medical Centre Ljubljana, who can be reached at peter.poredos@kclj.si.
